# Predicting the loss of organic archaeological deposits at a regional scale in Greenland

**DOI:** 10.1038/s41598-019-45200-4

**Published:** 2019-07-11

**Authors:** Jørgen Hollesen, Henning Matthiesen, Rasmus Fenger-Nielsen, Jakob Abermann, Andreas Westergaard-Nielsen, Bo Elberling

**Affiliations:** 1 Environmental Archaeology and Materials Science, The National Museum of Denmark, IC Modewegsvej, Brede, DK-2800 Lyngby, Denmark; 2 Center for Permafrost (CENPERM), Department of Geosciences and Natural Resource Management (IGN), University of Copenhagen, Øster Voldgade 10, DK-1350 Copenhagen, Denmark; 3 Asiaq, Greenland Survey, Postbox 1003, GL-3900 Nuuk, Greenland; 4 Department of Geography and Regional Science, Graz University, Heinrichstraße 36, 8010 Graz, Austria

**Keywords:** Environmental impact, Governance

## Abstract

Across the Arctic, microbial degradation is actively destroying irreplaceable cultural and environmental records that have been preserved within archaeological deposits for millennia. Because it is not possible to survey the many sites in this remote part of the world, new methods are urgently needed to detect and assess the potential degradation. Here, we investigate organic deposits at seven archaeological sites located along the dominating west-east climatic gradient in West Greenland. We show that, regardless of age, depositional history and environmental conditions, all organic deposits are highly vulnerable to degradation. A state-of-the-art model that simulates the effect of future climate change on degradation indicates that 30–70% of the archaeological fraction of organic carbon (OC) could disappear within the next 80 years. This range reflects the variation within the climatic gradient and the future climate scenario applied (RCP 4.5 and RCP 8.5). All archaeological deposits are expected to experience a substantial loss, but the most rapid degradation seems to occur in the continental inland areas of the region, dominated by dry and warm summers. This suggests that organic remains from the Norse Viking Age settlers are especially under threat in the coming years.

## Introduction

Archaeological sites in the Arctic contain extraordinarily well-preserved organic deposits that provide unique evidence of past humans^1–4^ and the ecosystems in which they lived^5–8^. Recent advances in analytical techniques including ancient DNA and isotope analysis have provided important new insight into population migrations^3,4^ and diet economy^9^ and thereby revealed the great potential of these rich archaeological deposits. This potential, however, is quickly disappearing due to climate change which increasingly damages archaeological sites and deposits^10–14^. Microbial degradation is considered one of the largest threats to the continued preservation of organic archaeological deposits^11–13^. Because the degradation rate is directly controlled by the soil temperature and moisture content^10,13^, rising air temperatures and changes in precipitation during the frost-free season may lead to a loss of organic key elements such as archaeological wood, bone and ancient DNA. Furthermore, the microbial degradation of organic deposits is accompanied by a significant heat production that may act as a potential positive-feedback mechanism that further increases soil temperatures and accelerates loss of organic carbon (OC)^13,15^. To date, quantitative estimates of current and future loss of OC in archaeological deposits have only been made for one site in the Arctic^10,15,16^ and little is therefore known about how, where and when sites are affected. Compared to other threats, such as coastal erosion, microbial degradation is more difficult to detect and monitor and, as a result, the loss of archaeological remains may progress unnoticed. With more than 180,000 archaeological sites registered in the Arctic^14^ it is impossible to visit and monitor all sites on a regular basis. Consequently, new methods to detect the most vulnerable sites are needed. Computer models have been used widely to assess the response of permafrost soils to climate change^17,18^ and to relate quantitatively the effect of changes in soil temperature and water content to the degradation of organic carbon (OC)^19,20^. However, because the physical and chemical compositions of archaeological deposits are markedly different from those of natural soils^21^, their long-term vulnerability cannot be assessed using such projections.

Here we investigate the physical and chemical properties of seven different organic archaeological deposits in the Nuuk region in Southwest Greenland and use the results to develop a model setup that can be used to predict current and future soil temperatures, water content and loss of OC. Based on results from previous degradation studies of organic archaeological materials^10,13,15^, we focus on the oxic degradation of organic deposits that contain important residues of human subsistence and in which various types of artefacts are embedded.

## Results and Discussion

### Study sites and environmental conditions

**Figure 1:**
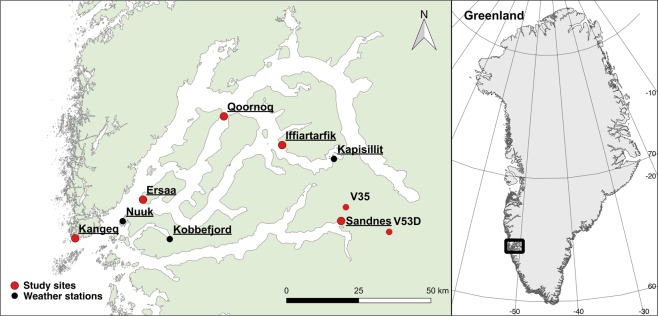
Study site locations. The seven study sites (red dots) are located in the Nuuk region along the dominating west-east climatic gradient in West Greenland^40^. Automated weather stations were installed at five key sites (underlined text). In addition, data from three official meteorological stations were used (black dots). Figure 1 was generated by Rasmus Fenger-Nielsen in QGIS 2.14 (https://www.qgis.org/da/site/), using the Layout View panel.

The seven archaeological sites are located along a transect stretching from the sea in the West and approx. 120 km inland towards the Inland Ice Sheet to the East (Fig. 1). They originate from the three main cultures of Greenland: Saqqaq (2,500–800 BC), Dorset (300BC–600AD), and Thule (1,300AD–present), as well as from the Norse Viking Age settlers who inhabited the area from approximately 985–1,350 AD^22–24^ (Supplementary Table S1). At all sites, the archaeological deposits are found in the uppermost section of the subsurface, capped only by a 5–10 cm turf layer.

**Figure 2:**
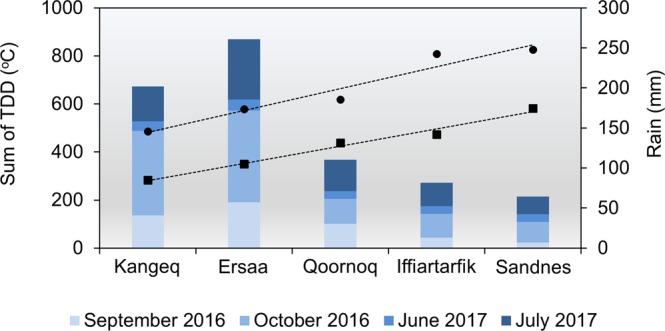
Climatic variations in the study region. Observed precipitation rates in the frost-free period (bars) and sum of thawing degree days (TDD, dashed lines) from 1^st^ September 2016–31^st^ July 2017 derived from air temperatures (circles) and soil temperatures in 0.1 m depth (squares).

To investigate climatic variations in the study region, automated weather stations were installed at five key sites (Fig. 1). Results show that the sites are subject to very different weather conditions, with inland sites having almost twice as many thawing degree days (TDD) as sites at the outer coast. Inland sites received significantly less rain (Fig. 2). The observed variations in precipitation are reflected in soil water contents during the summer. Upper soil layers become drier with increasing distance from the outer coast (Supplementary Fig. S1). Comparison with thawing degree days derived from the MODIS-based land surface temperature (LST) product MOD11A1 V6 show that the variation observed across the sites during the study period represents the variation seen in the region from 2001–2017 (Supplementary Fig. S2).

### Characteristics of the archaeological deposits

Soil pits were made at all study sites to obtain soil samples for measuring the physical and chemical properties, as well as the degradability of the archaeological deposits. Measurements of porosity, loss on ignition and soil bulk density were conducted on a total of 22 samples from seven sites (Supplementary Fig. S3). The site mean porosities ranged from 71–82 vol.% with a total mean = 77.1 ± 2.9 vol. % (±1 s.d., n = 7), the site mean loss on ignition varied between 18 and 39 wt% dw^−1^ with a total mean = 30.3 ± 6.5 wt% dw^−1^ (±1 s.d., n = 7) and the site mean bulk densities varied from 254–673 kg m^−3^ with a total mean = 458 ± 120 kg m^−3^ (±1 s.d., n = 7). Loss on ignition and bulk densities showed considerable variations within and across sites (Supplementary Fig. S3).

The thermal conductivity (*kh*) and heat capacity (*hc*) were measured *in situ* (unfrozen conditions) at the five key sites (Supplementary Fig. S4). The variation of *kh* and *hc* was just as much within study sites as between sites, with variations significantly positively correlated to the soil water content (linear regression for *kh*: r^2^ = 0.70, p < 0.01, n = 46; linear regression for *hc*: r^2^ = 0.68, p < 0.01, n = 46) (Supplementary Fig. S4).

**Figure 3:**
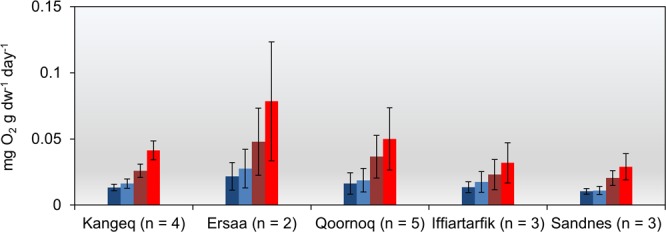
Oxygen consumption during incubation. Observed oxygen consumption rates in 17 different samples at 1 °C (dark blue), 5 °C (light blue), 10 °C (dark red) and 15 °C (light red). The n-values represent the number of different depth-specific samples investigated. Vertical bars show ±1 s.d.

The degradation potential of the archaeological deposits was investigated using the oxygen consumption method^25^. Measurements were made on 17 depth-specific samples from the five key sites at 1, 5, 10 and 15 °C (Fig. 3 and Supplementary Fig. S5). This was done in four separate runs from low to high temperatures with a total incubation time of 45 days. Site mean rates at 5 °C varied between 0.01 and 0.03 mg O_2_ g dry soil^−1^ day^−1^ with a total mean = 0.018 ± 0.005 mg O_2_ g dry soil^−1^ day^−1^ (mean ± 1 s.d., n = 5) (Fig. 3 and Supplementary Fig. S5). Oxygen consumption rates showed considerable variations within and across sites. A significant increase in reactivity with increasing temperature was observed (Fig. 3; Supplementary Table S2). The increase in oxygen consumption per 10 °C temperature change (the Q_10_ value) varied between 1.5 and 2.7 with a mean value of 2.3 ± 0.4 (±1 s.d., n = 17) which agrees with previous studies of both organic archaeological deposits and permafrost-affected soils^13,15,26^.

### Modelling current conditions

**Figure 4:**
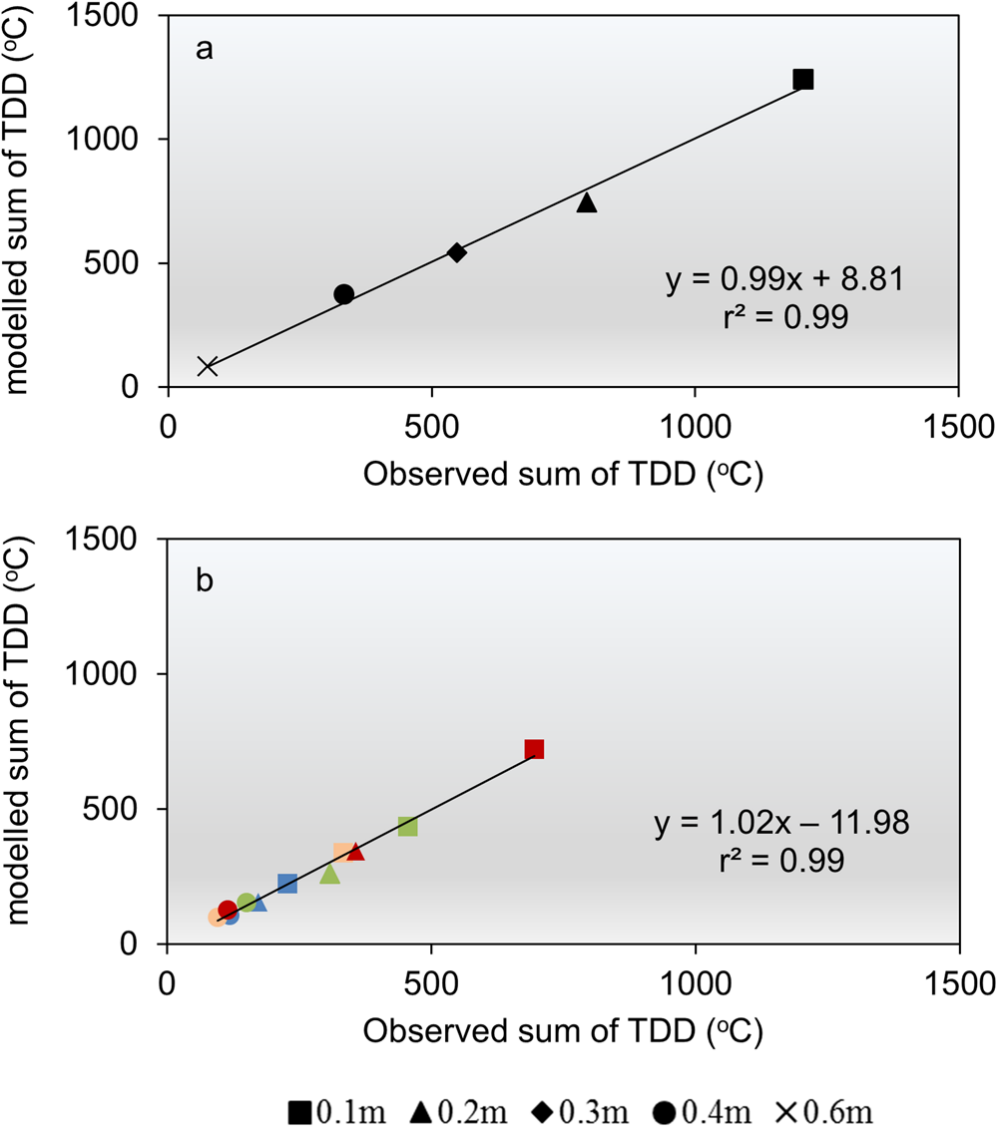
Observed versus modelled TDD. (**a**) At the site of Kangeq from 1^st^ January 2015–31^st^ August 2017. (**b**) At the sites Ersaa (blue) and Iffiartarfik (light red) for the period 1^st^ June to 31^st^ July 2017 and for the sites Qoornoq (green) and Sandnes (red) for the period 1^st^ June to 31^st^ August 2017.

The thermal and hydrological regimes of the five key sites were simulated using the well-established heat and water flow model, the CoupModel^27^. This model has previously been used to investigate the importance of microbial heat production on permafrost thawing and degradation of soil organic carbon (SOC) in Arctic soils, including an organic archaeological deposit^15^. The characteristics of the archaeological deposits were modelled based on the site-specific observations of porosity, bulk density and total organic content; the total mean of all samples from all sites was applied. Mean daily air temperatures and precipitation rates were used as meteorological input in the model. Air temperature equivalents were derived from the MODIS-based land surface temperature product MOD11A1 V6, in agreement with previous work^28^. The model was initially calibrated for 2.5 years of measured soil temperatures and water contents at the site of Kangeq (1^st^ January 2015–31^st^ August 2017) (Supplementary Figs S6 and S7). Measured summer soil temperatures and water contents (2017) from the four remaining key sites were used to test the model (Supplementary Figs 8–10 Initially, this was done after adjusting only the meteorological input data to site-specific conditions. However, the soil thermal conductivity proved to be a crucial parameter that had to be calibrated separately for each site. High r^2^ values (0.55–0.97) and low mean differences (−0.65–0.19 °C) for both the calibration and test runs show that the model can describe variations in the overall temperature regimes of the different sites (Supplementary Table S3). The model also represents accurately the amount of observed TDD (Fig. 4), and confirms its overall robustness to describe the amount of energy available during the frost-free period. No significant differences (p = 0.39; paired t-test) are seen between simulated and observed values of soil water contents (Supplementary Fig. S9). However, compared to the simulations of soil temperatures, there is a larger discrepancy between simulated and observed values of soil water, especially for the absolute soil water content (Supplementary Fig. S10).

### Predicting future conditions and the loss of organic archaeological deposits

The CoupModel was used to investigate the effects of future climate warming on soil temperatures at the five study sites. This was done by simulating two different climate change scenarios (Table 1) based on the mean predictions from the regional climate model RCM HIRHAM5^29,30^ driven with RCP 4.5 and RCP 8.5 greenhouse gas concentration scenarios (IPCC)^31^. A three-pool degradation model was included in the CoupModel to investigate the effects of changes in soil thermal and hydrological conditions on the degradation of organic carbon (OC). Based on terminology and values used in other studies^32–34^, the total pool of OC was divided into a fast (5%), a slow (50%), and a passive pool (45%). The fast pool was considered to represent new OC from fresh plants and animal residues, the slow pool old OC from both archaeological and natural sources and the passive pool highly degraded and no longer biologically active OC. The total observed degradation rate derived from the oxygen consumption measurement was considered to be a sum of three pool-specific rates with turnover times under drained conditions at 5 °C of 1, 50 and >4,000 years, respectively. The absolute loss of carbon was calculated for each soil layer in 24 hour steps and adjusted to the depth-specific temperatures and water contents represented by specific climate change scenarios (see methods section).

**Table 1:** Future climate scenarios.

Temperature increase (°C)	RCP4.5	RCP8.5
Yearly	2.5	5.0
Winter (DJF)	3.1	6.0
Spring (MAM)	2.5	5.0
Summer (JJA)	2.0	3.9
Autumn (SON)	2.5	5.0

The two climate change scenarios used in the CoupModel to predict future soil temperatures were based on RCM HIRHAM5 driven with RCP 4.5 and 8.5 greenhouse gas concentration scenarios. The increase in air temperature by 2100 was relative to the 2000–2017 mean.

**Figure 5:**
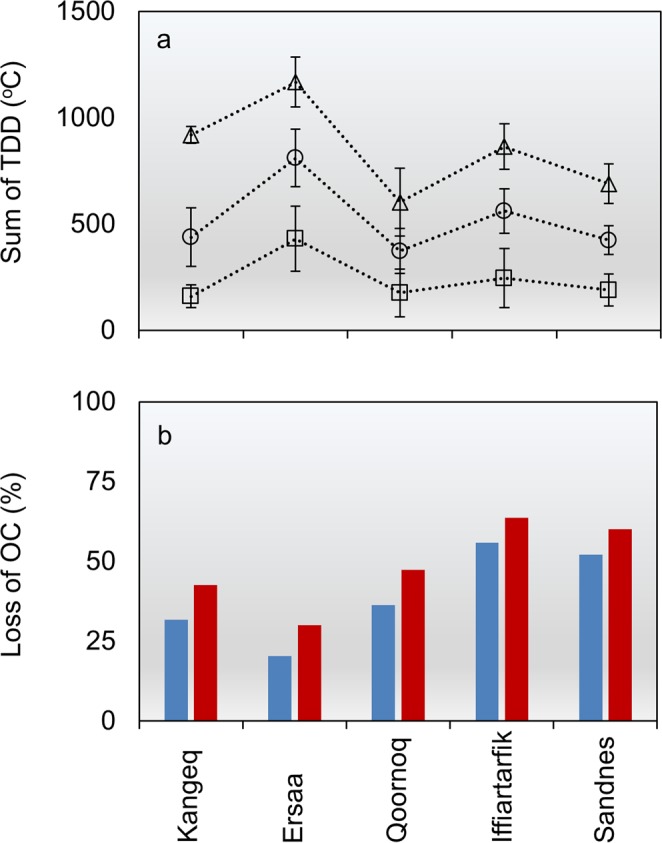
Model results. (**a**) Modelled sum of TDD in 0.4 m depth at the five key sites under current climatic conditions (squares), RCP 4.5 (circles) and RCP 8.5 (triangles). (**b**) Percentage loss from 2017–2100 under RCP 4.5 (blue) and RCP 8.5 (red) of the slow OC pool containing the archaeological OC fraction.

The results show that a 2.5 °C (RCP 4.5) and 5.0 °C (RCP 8.5) warming from 2017–2100 could increase mean annual temperatures at the five sites (0.4 m depth) by 0.4–1.0 °C and 1.2–2.6 °C respectively. Depending on the climate change scenario, this will increase the amount of TDD with 196–379 °C or 425–760 °C (Fig. 5) and prolong the duration of the frost-free period with 23–75 days or 53–104 days. Currently 0.3–0.8% of the slow OC pool (containing the archaeological OC fraction) is lost per year at 0.4 m. If temperatures increase as predicted, the loss may increase to 0.5–1.1% year^−1^ (RCP 4.5) and 0.8–1.6% year^−1^ (RCP 8.5). As a result, 20–63% of the current slow OC pool in 0.4 m could be lost within the next 80 years (Fig. 5). When integrated over the upper 0.5 m of the deposits for the same time period, results show that 30–70% of the slow OC fraction could be lost. The most rapid degradation appears to occur in the relatively dry continental inland parts of the study region, where many important remains of the Norse Viking Age settlers are found^22–24^. Here a loss of more than 35% within the upper 0.5 m may occur over the next 30 years. These values represent the loss of the current slow OC pool in the deposit. Although in an ecosystem context, this loss will be partly offset or maybe even exceeded by a similar carbon uptake by growing plants^35^_,_ this has little relevance for the loss of significance of archaeological remains. Because very limited knowledge is available concerning the long-term degradation of archaeological materials and artefacts, it is not possible to estimate how long it will take to destroy remains of for example human hair, bone collagen or DNA. However, we expect the loss of OC in the organic deposits to be closely linked to a loss in quality of buried organic artefacts^10,12^ and that this loss of information is a continuous process. Therefore, the scientific potential of these remains may be reduced significantly before all the archaeological OC has been degraded.

Previous studies of organic archaeological deposits at the site Qajaa, located 550 km north of Nuuk, have shown that permanently frozen archaeological deposits produce substantial quantities of heat when thawed/decomposed^15^. We performed our simulations with and without heat production and found that the warming effect from microbial heat production was negligible. The degradation rates observed in the Nuuk area are on average 3 times slower than those at Qajaa, and the Nuuk deposits are much thinner (<1 m compared to 3 m). This explains the low heat production potential and confirms that the archaeological deposits in the “warmer” Nuuk area are already significantly degraded.

### Implications and future investigations

The model applied here represents considerable progress in developing methods to pinpoint vulnerable sites in the Arctic. Despite the studied deposits varying greatly with respect to origin, age and exposure to different environmental conditions, it was possible to describe the thermal and hydrological characteristics of the deposits using total mean values (mean of all samples from all sites) of porosity, bulk density and total organic content. This, combined with the fact that it was possible to model soil temperatures and the TDD accurately based on globally-available MODIS data (available at 1 × 1 km resolution since 2001), is very promising. However, the projections also reveal considerable uncertainties, especially in relation to modelling the soil water content. As is the case for most parts of the Arctic, no reliable model for regional precipitation and snow currently exists for the study region. Instead, we used data calculated from regressions between site-specific measurements and two official meteorological stations in the area. While producing the best available data, this highlights some uncertainties, especially in the Arctic where local precipitation patterns, wind, topography and aspect may have a great influence on precipitation rates and on snow depths. As a result, thermal conductivity had to be calibrated separately for each site in the model to compensate for uncertainties in soil water. The subsequent simulations were made with current precipitation rates, but using a dynamic snow model. For the Nuuk area, it is predicted that precipitation rates will increase by approx. 10% from 2017 to 2100 while evapotranspiration and runoff are also likely to increase^30^. We acknowledge that the present-day water balance and vegetation cover may not represent the future accurately.

One major limitation of the model applied is that it only considers degradation of OC. Although we expect the loss of OC to reflect a loss of organic artefacts, the actual rates of degradation of different types of archaeological materials/artefacts are unknown. Future degradation experiments should therefore include a broad spectrum of organic archaeological materials and artefacts. This would provide the fundamental knowledge needed to improve the model to include differential degradation patterns and ultimately help to predict the time period at which different types of information are lost.

Almost 6000 archaeological sites are currently registered in Greenland out of which ~250 are located in our study region. Our results highlight that organic deposits at many of these sites may be damaged or partly lost as soil temperatures and water contents change within the next century. The methods described here may help to identify particularly vulnerable sites and thereby help to direct limited resources to where they are most needed. We acknowledge that this is only a step towards saving these important records of the past. So far, very little has been done to develop methods for mitigation of archaeological sites in the Arctic, and therefore excavation is the only solution for rescuing archaeological remains at risk. Excavations are, however, expensive and time-consuming, and there are no designated funds or programmes for archaeological rescue excavations. Thus, we must also be realistic and acknowledge that it will be necessary to prioritise sites in order to focus limited resources on the most valuable sites.

## Methods

### Meteorological conditions

Monitoring equipment was installed in 2016 at five sites to investigate the site-specific meteorological conditions. An overview of those environmental parameters monitored and the equipment used is given in Supplementary Table S4.

### Soil-physical and chemical properties

Test pits excavated at each site. Thermal properties (KD2 Pro instrument from Decagon devices, INC., Pullman, WA, USA) were measured vertically at 5–20 cm increments in the test pits. Volume specific soil samples (100 cm^3^), as well as larger bulk samples were taken from distinct layers and were kept frozen during transport to Denmark. The volume specific samples were used to determine the loss on ignition (460 °C/16 hours), water content, soil porosity and soil bulk density.

### Incubation experiments

The collected soil bulk samples were homogenized manually (stones, larger bones, and wooden fragments were first removed) and triplicates were extracted and used for measurements of O_2_ consumption. The triplicates were placed in vials, which were sealed using a disc of transparent plastic commercial oxygen barrier film (EscalTM), a silicone gasket and a screw cap with an aperture. Oxygen sensor foil (SF-PSt5-1223-01, PreSens) was glued to the inside of the oxygen barrier film, and the OxoDish® readers were placed on top of the vials. The decrease of headspace O_2_ concentrations with time (7–19 days) was measured for each sample using SDR SensorDish® readers (PreSens Precision Sensing GmbH, Regensburg, Germany). Measurements were first made at 1 °C and then at 5, 10 and 15 °C. Between each run the vials were flushed with atmospheric air. Samples were incubated for 45 days in total. The measured oxygen consumption rates (mg O_2_ g dry soil^−1^ day^−1^) were used to calculate the basal soil respiration rate (BSR) assuming that one mole of oxygen can oxidise one mole of organic carbon.

### Modelling site specific soil temperatures and soil moisture

The CoupModel model was first calibrated to match 2.5 years (1^st^ January 2015–31^st^ August 2017) of measured soil temperatures and water contents at four different depths at the site of Kangeq (Supplementary Figs S6 and S7). Subsequently, the model was tested based on measured soil temperatures and soil water contents (1^st^ June –31^st^ July 2017) from the four remaining key sites (Supplementary Figs 8–10 This was done after adjusting only the meteorological input data and calibrating the thermal conductivity to site-specific conditions.

To avoid any initial instability in soil temperatures and soil water contents, all simulations were initiated 1^st^ January 2010. Mean daily air temperatures and precipitation were used as meteorological input in the model. Air temperature equivalents were derived from the MODIS-based land surface temperature product MOD11A1 V6, in agreement with previous data^28^ that was gap-filled with data from MAR3.7^36^. The validity of the air temperature input was tested via linear regressions with site specific observations from 1^st^ September 2016–31^st^ July 2017 (Supplementary Fig. S11). We used precipitation data from two meteorological stations located in Nuuk and Kapisillit (Fig. 1) that are run by ASIAQ (Greenland Survey). The validities of the precipitation rates were tested via linear regressions with site specific observations from 1^st^ September 2016–31^st^ July 2017 (Supplementary Fig. S12).

The surface temperature was calculated using an equation suggested by Brunt^27^ based on the air temperature. When the ground was covered by snow, the surface temperature was adjusted by a weighting factor based on thermal conductivities of the upper soil layer and the snow density in addition to the thickness of both layers. Snow was modelled by assuming that precipitation consisted only of snow when air temperatures were below 0.5 °C. The duration of the snow cover season was adjusted based on images captured daily by automatic cameras installed at three of the sites. As no snow depth measurements were made at the study sites, we tested the snow model using data from the Nuuk Basis research station^37^ located in the nearby Kobbefjord (Fig. 1 and Supplementary Fig. S13). To account for local variations at the sites such as snow drift by wind, winter precipitation rates were adjusted based on discrepancies between simulated and observed surface temperatures (Supplementary Fig. S14).

The model regime consisted of a 5.0 m deep profile divided into 64 layers. The upper 1.0 m (21 layers) were described by site-specific and depth-specific observations; mean values were applied across the five sites. The soil below 1.0 m was considered similar to the entisol/cryosols normally found in this area of Greenland^38^. Water uptake by plants and soil evaporation was treated as a common flow from the uppermost soil layers. The relative distribution of water uptake from soil layers was based on measured depth specific root distribution, default values of potential transpiration rates (4 mm day^−1^) and a growing season of 140 days based on observation from the Nuuk Basic research station^38^.

Water retention capacities and hydraulic conductivities were estimated from the measured porosity and organic content (Supplementary Fig. S3) using the Mualem and Brooks & Corey equations. The heat capacity (*hc*) and thermal conductivity (*kh*) were calculated as functions of soil solids and soil moisture. For unfrozen conditions this function was based on measured values *hc* and *kh*. (Supplementary Fig. S3). For frozen conditions, default values for organic soils were used. For the layers below the archaeological deposits values of *hc* and *kh* were based on default values for mineral soils.

### Modelling carbon degradation

A carbon degradation module was used in the CoupModel to estimate the degradation of OC with time.

Total modelled soil respiration R was the sum of three pool-specific degradation rates (*ki*), which were each calculated according to Equations 1 and 2.

1 $$R={\sum }_{i=1}^{3}\,{r}_{i}={\sum }_{i-1}^{3}\,{k}_{i}O{C}_{tot}\,{f}_{i}$$

2 $${f}_{i}=\frac{O{C}_{i}}{O{C}_{tot}},{\sum }_{i=1}^{3}\,{f}_{i}=1$$

where O*C*_*tot*_ was the initial OC pool, *f*_*i*_ a fractionation coefficient that describes the ratio of the current OC pool (OC*i)* to the total C pool. The total pool of OC was divided into three subpools; a fast (5%), a slow (50%) and a passive pool (45%)^32–34^. The turnover times for each of the pools (under drained conditions at 5 °C) corresponded to 1, 50 and >4,000 years respectively^32,33^. We considered the archaeological fraction of OC to be in the slower degrading pool. The absolute loss of carbon was calculated in 24 hour steps for each soil layer and adjusted to the simulated depth-specific temperatures using a Q_10_ value of 2.3 (the average of measured values) and soil moisture using a previously reported sensitivity function^27^. The effect of microbial heat production was tested by performing simulations with and without heat production. The heat production was applied by assuming that one mole of carbon release 40 MJ kgC^−1^ as previously proposed^39^.

## Supplementary information

Supplementary information

## Data Availability

The datasets generated during and/or analysed during the current study are available from the corresponding author on reasonable request.
