# Supplementary material for: Predicting the loss of organic archaeological deposits at a regional scale in Greenland
Source: Sci Rep. 2019 Jul 11;9:9097. doi: 10.1038/s41598-019-45200-4 (PMC6624202; doi:10.1038/s41598-019-45200-4)
Supplement: Supplementary file 1 — Supplementary information [file 41598_2019_45200_MOESM1_ESM.pdf]

Supplementary information:

Predicting the loss of organic archaeological deposits at a regional scale in Greenland

Jørgen Hollesen[1,2], Henning Matthiesen[1], Rasmus Fenger-Nielsen[1,2], Jakob Abermann[3,4], Andreas Westergaard-Nielsen[2] & Bo Elberling[2]

[1] *Environmental Archaeology and Materials Science, The National Museum of Denmark, IC Modewegsvej, Brede, DK-2800 Lyngby, Denmark*

[2] *Center for Permafrost (CENPERM), Department of Geosciences and Natural Resource Management (IGN), University of Copenhagen, Øster Voldgade 10, DK-1350 Copenhagen K, Denmark*

[3] *Asiaq, Greenland Survey, Postbox 1003, GL-3900 Nuuk, Greenland*

[4] *Department of Geography and Regional Science, Graz University, Heinrichstraße 36, 8010 Graz, Austria*

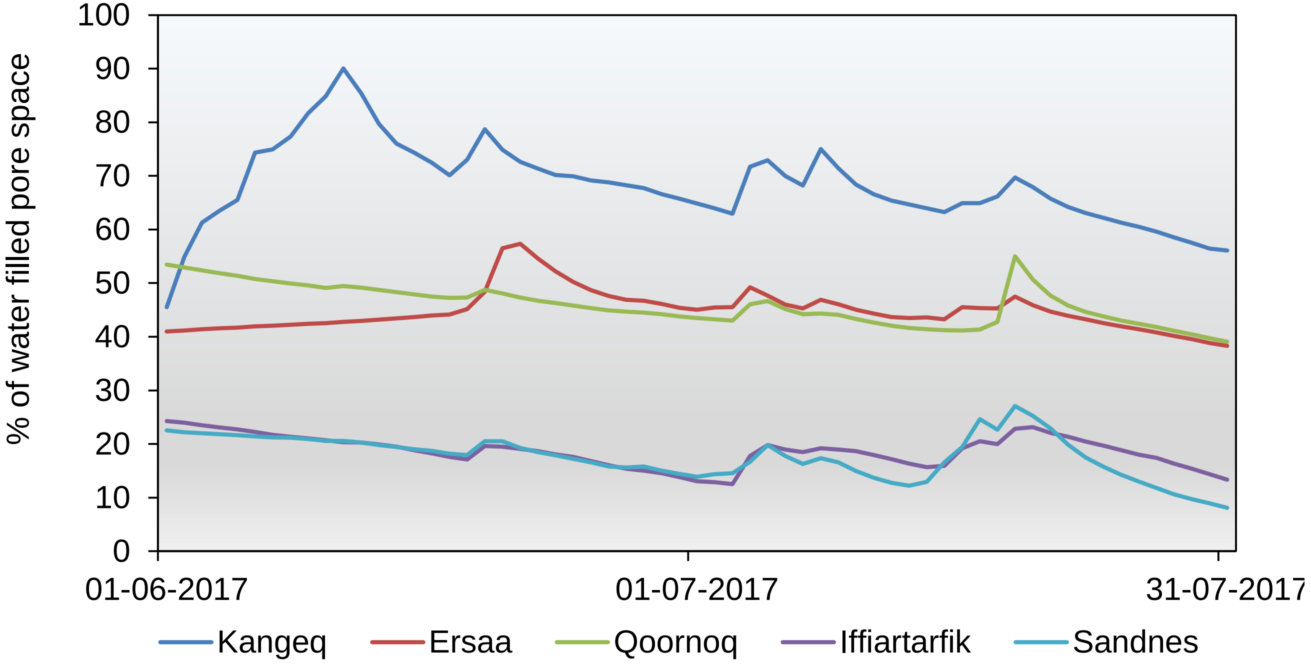

Supplementary Fig. S1: Percentage of water filled pore space in 0.1 m depth at the five study sites during the summer 2017. The percentage of water filled pore space is the ratio of volumetric soil water content to total soil porosity.

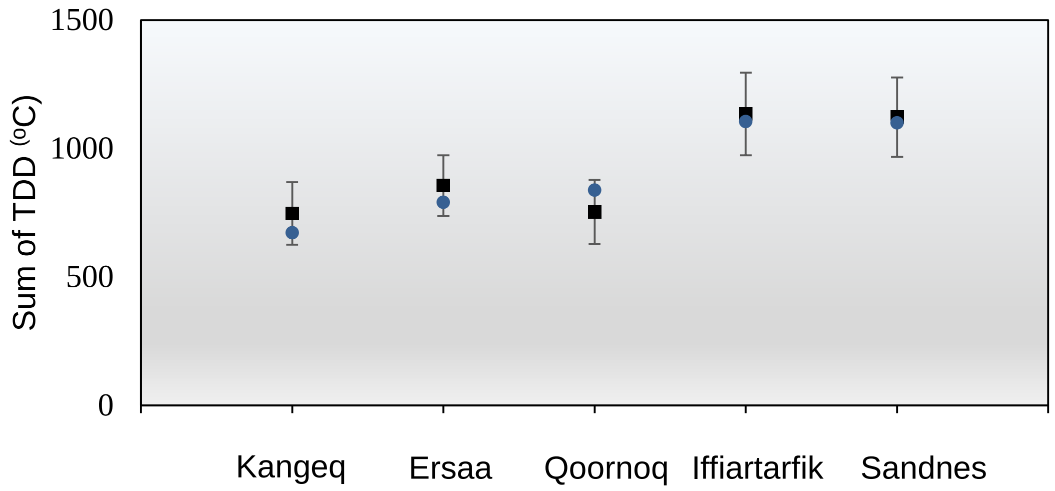

Supplementary Fig. S2: Mean annual sum of TDD for the period 2001–2017 derived from the MODIS-based land surface temperature product MOD11A1 V6 (black squares) and observed at the study sites from 1 September 2016–31 August 2017 (blue circles). Vertical bars show ± 1 s.d.

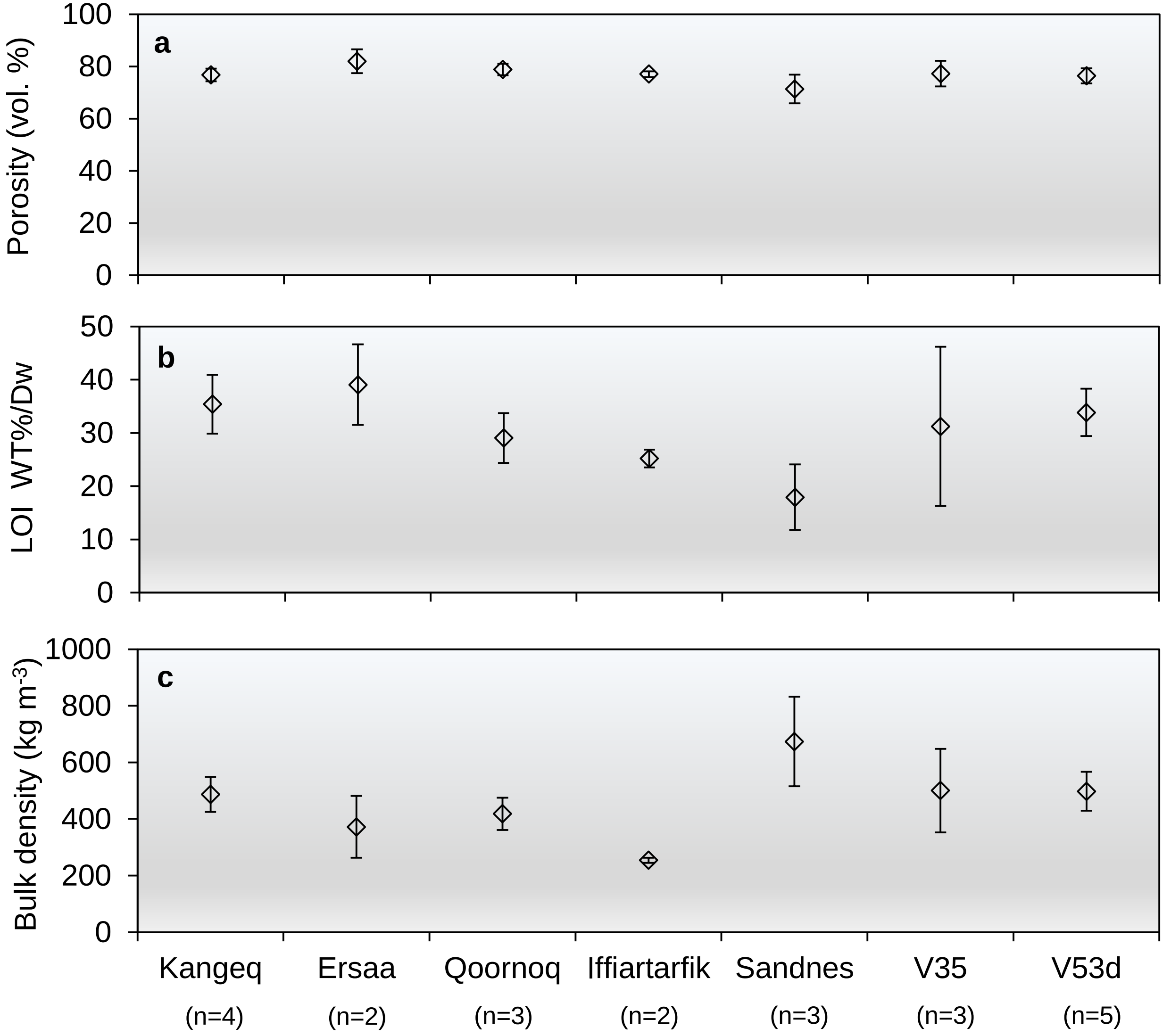

Supplementary Fig. S3: Observed mean values of porosity (a), organic content (b) and bulk density (c). Vertical bars show ± 1 s.d.

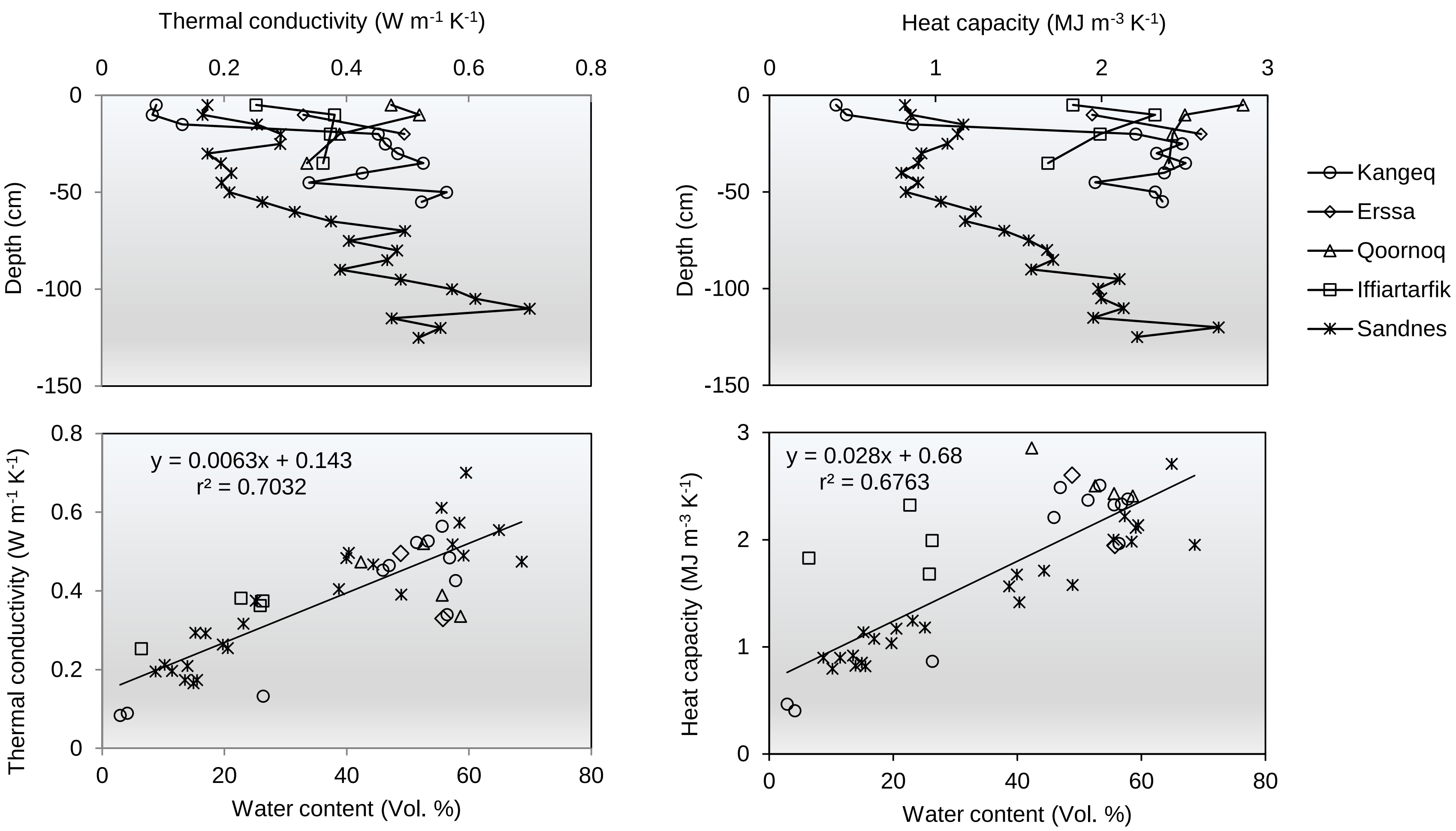

Supplementary Fig. S4: Upper: observed depth-specific values of thermal conductivity (left) and heat capacity (right). **Lower:** Linear regression between observed ground water contents and values of thermal conductivity (left) and heat capacity (right).

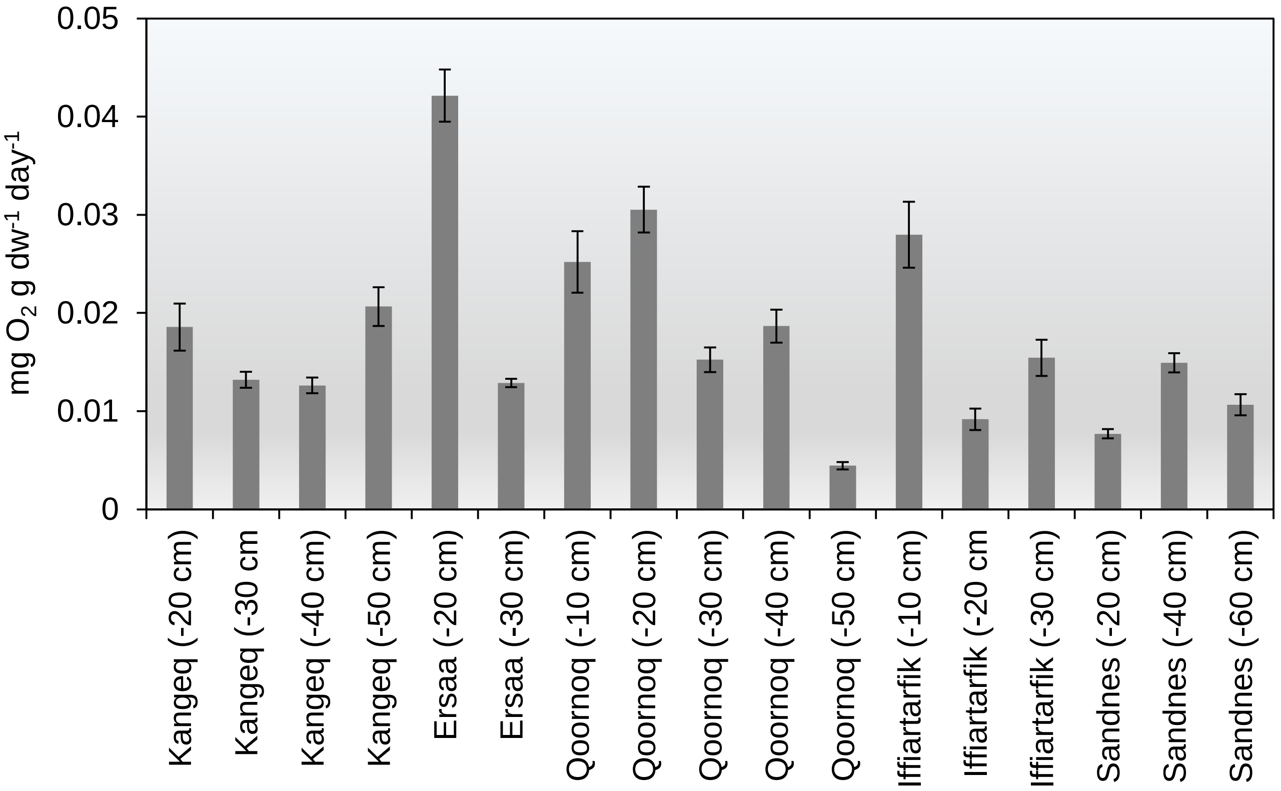

Supplementary Fig. S5: Observed oxygen consumption rates in 17 different samples at 5 °C. Vertical bars show ± 1 s.d. (n =3).

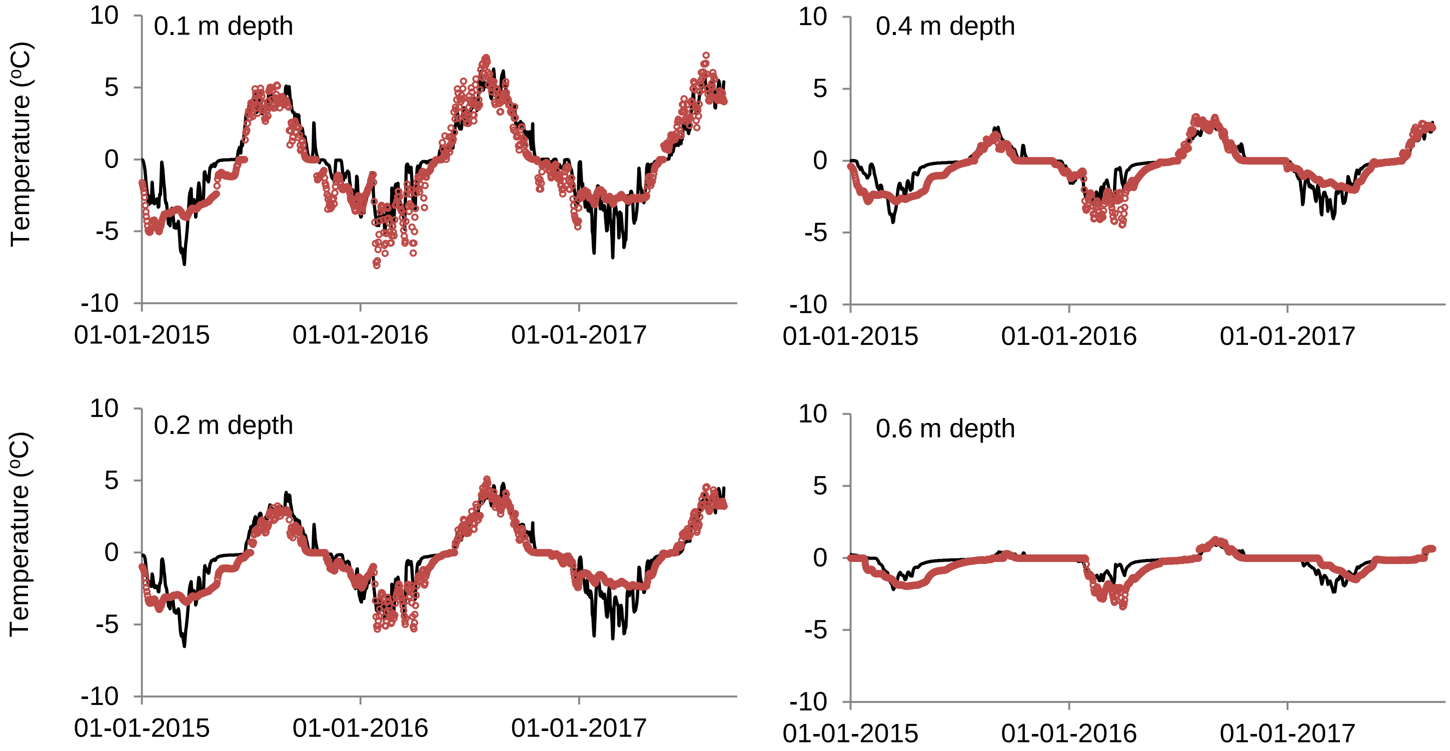

Supplementary Fig. S6: Simulated (red) and measured (black) ground temperatures at the archaeological site Kangeq from 1st January 2015–31st August 2017.

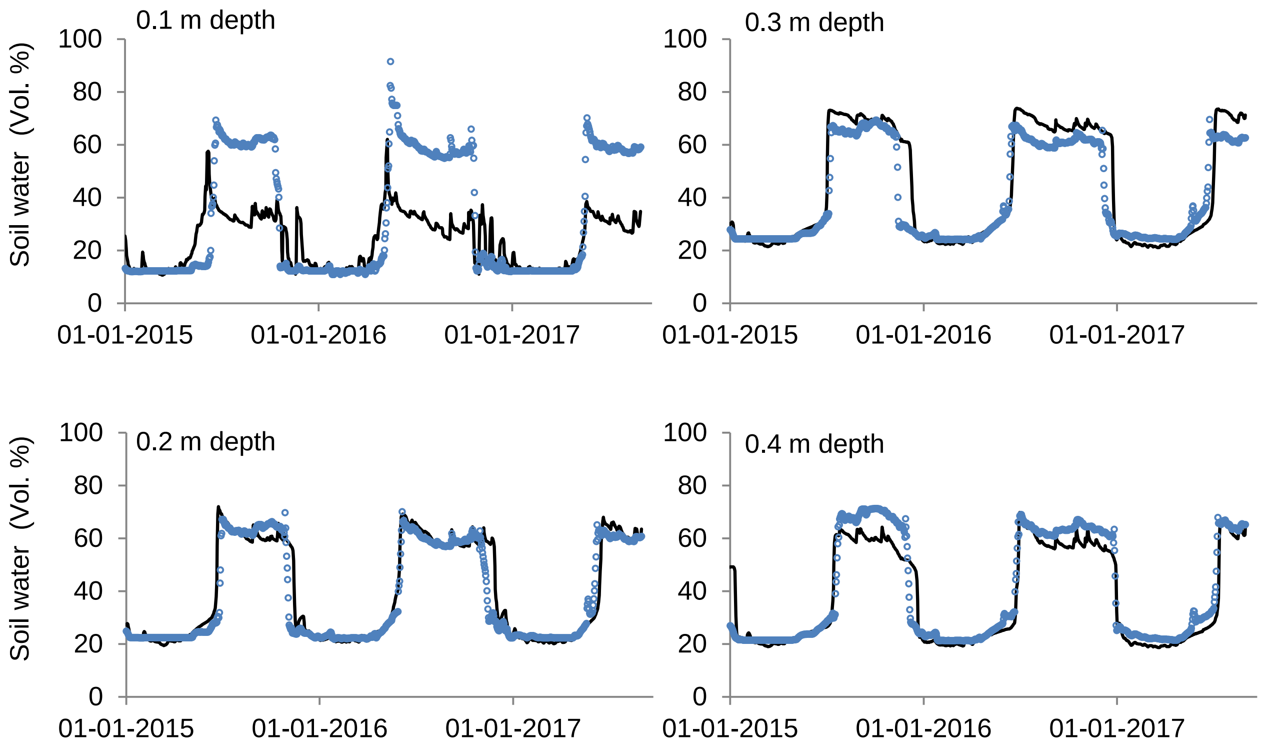

Supplementary Fig. S7: Simulated (blue) and measured (black) soil water contents in the archaeological deposits at the site Kangeq from 1st January 2015–31st August 2017. The low water content during the winter periods is due to soil frost.

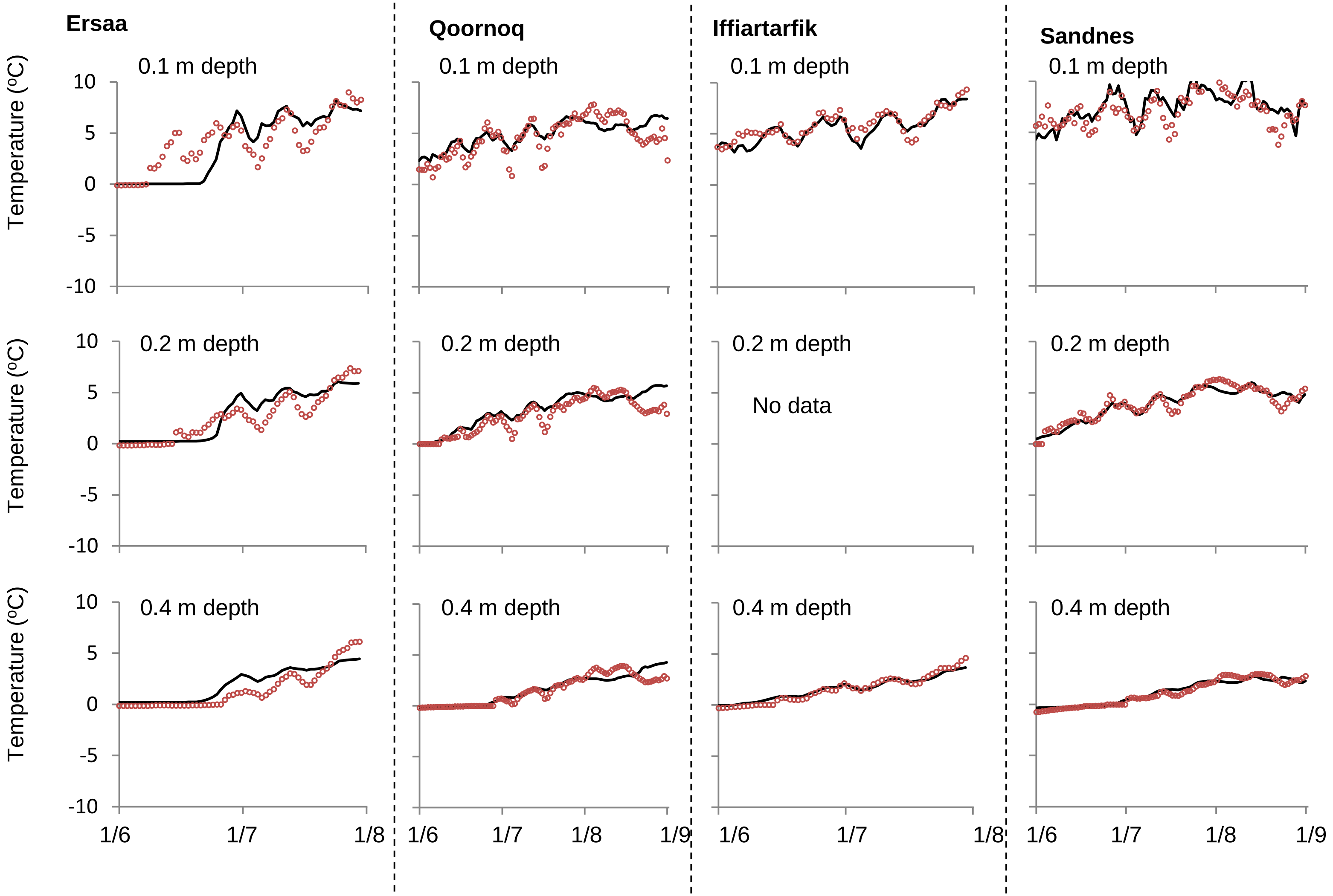

Supplementary Fig. S8 Simulated (red) and measured (black) soil temperatures at the archaeological sites Ersaa, Qoornoq, Iffiartarfik and Sandnes during the summer period 2017.

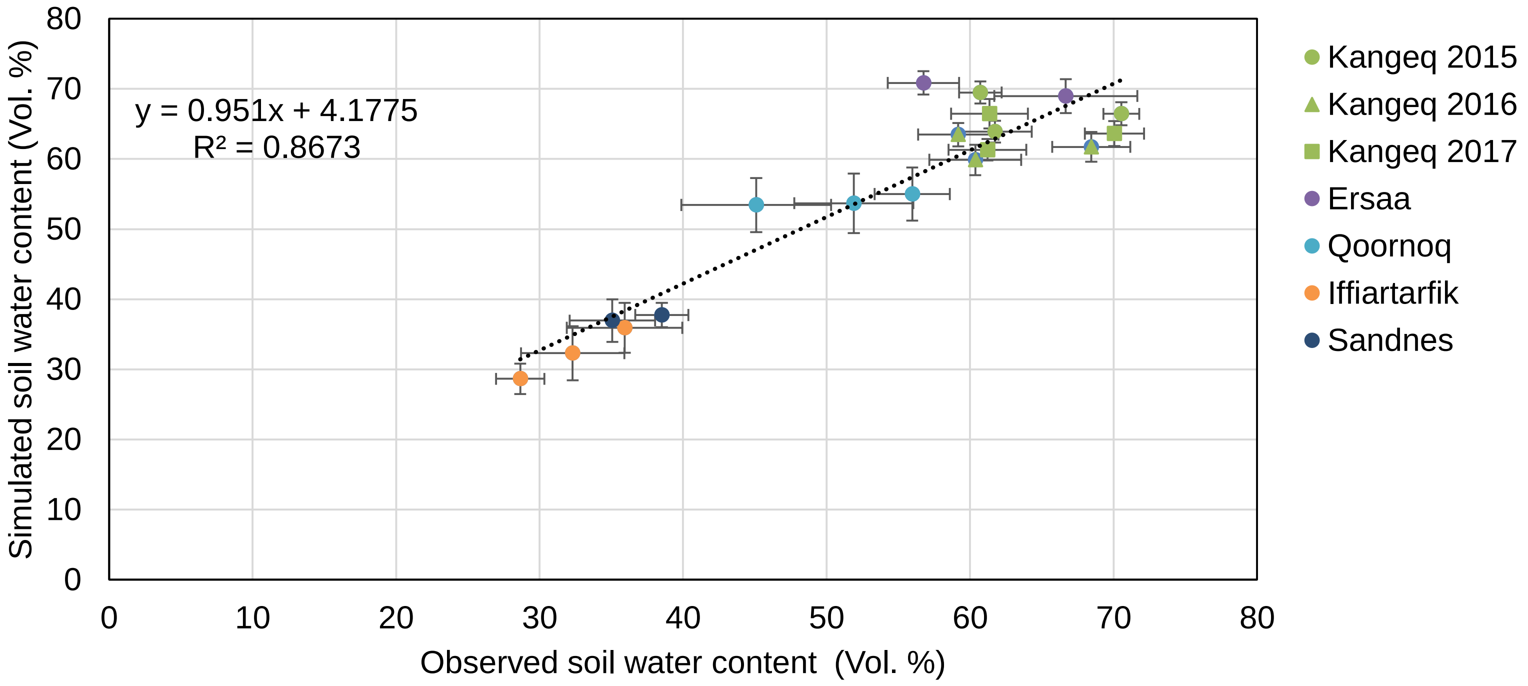

Supplementary Fig. S9: Linear regression between mean simulated and mean observed ground water contents for the frost free summer period. Vertical and horizontal bars show ± s.d.

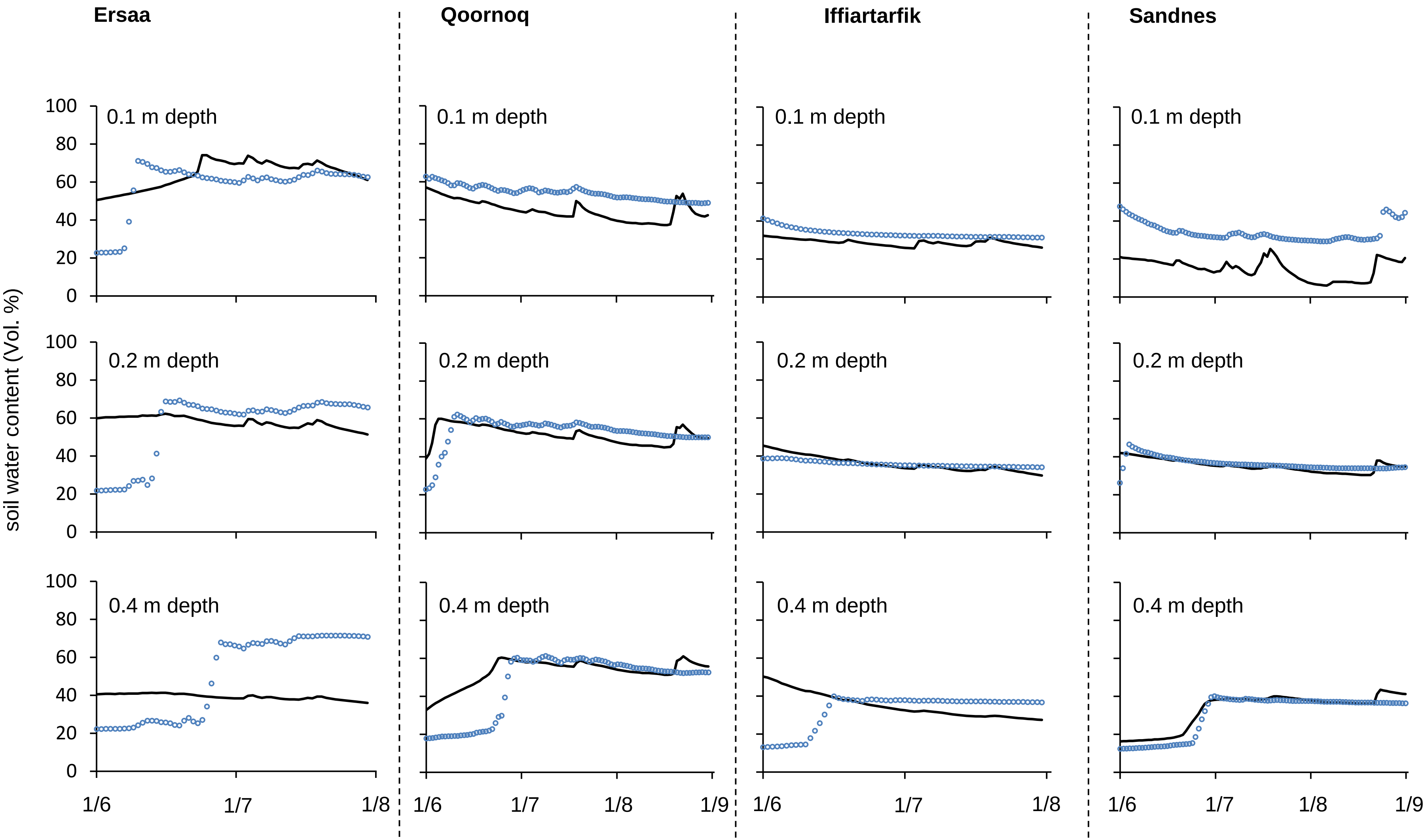

Supplementary Fig. S10: Simulated (blue) and measured (black) soil water contents at the archaeological sites Ersaa, Qoornoq, Iffiartarfik and Sandnes during the summer period 2017.

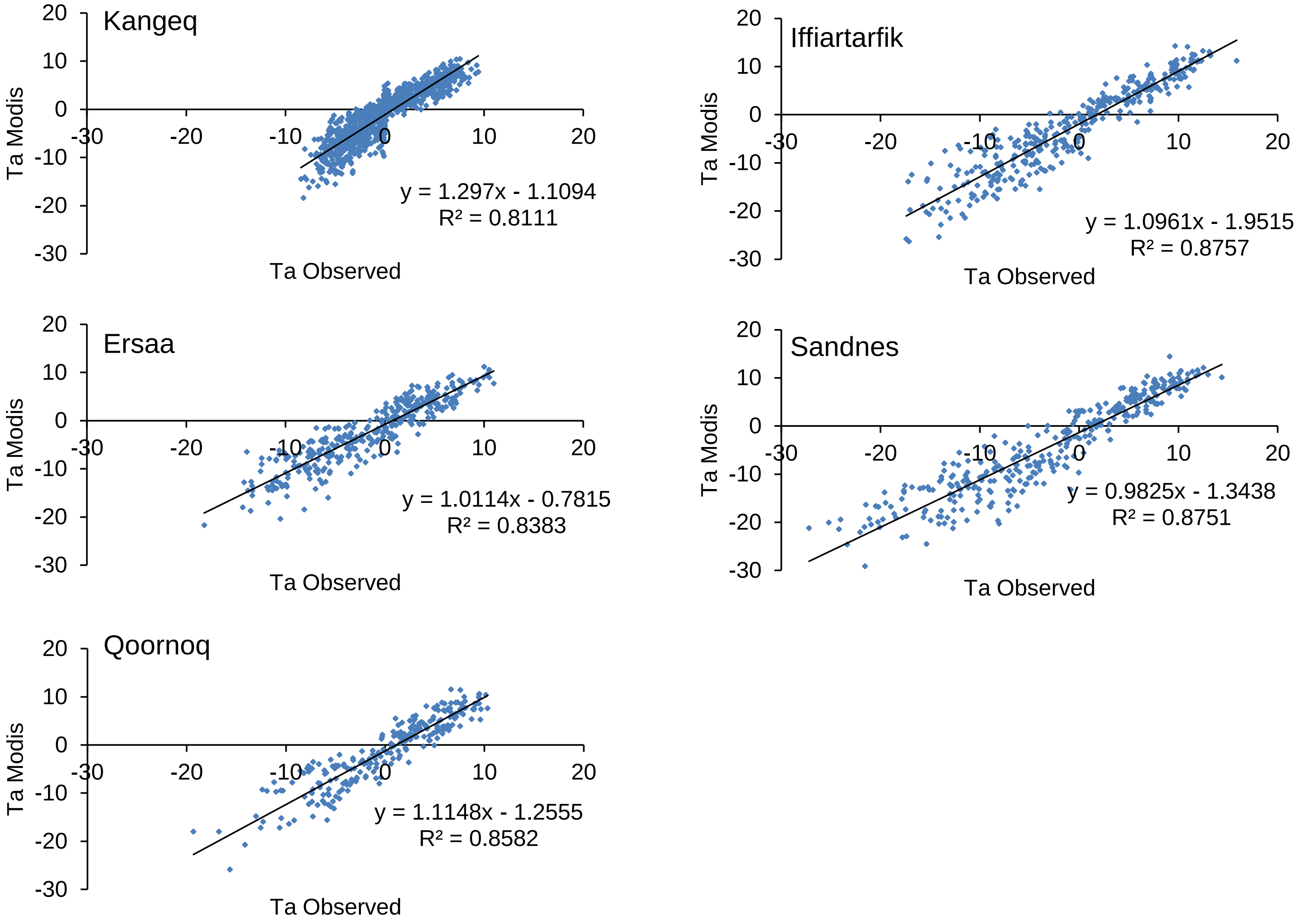

Supplementary Fig. S11: Linear regression between observed air temperatures (Ta) and air temperatures derived from MODIS-based land surface temperature product MOD11A1 V6 that was gap-filled with data from MAR3.7.

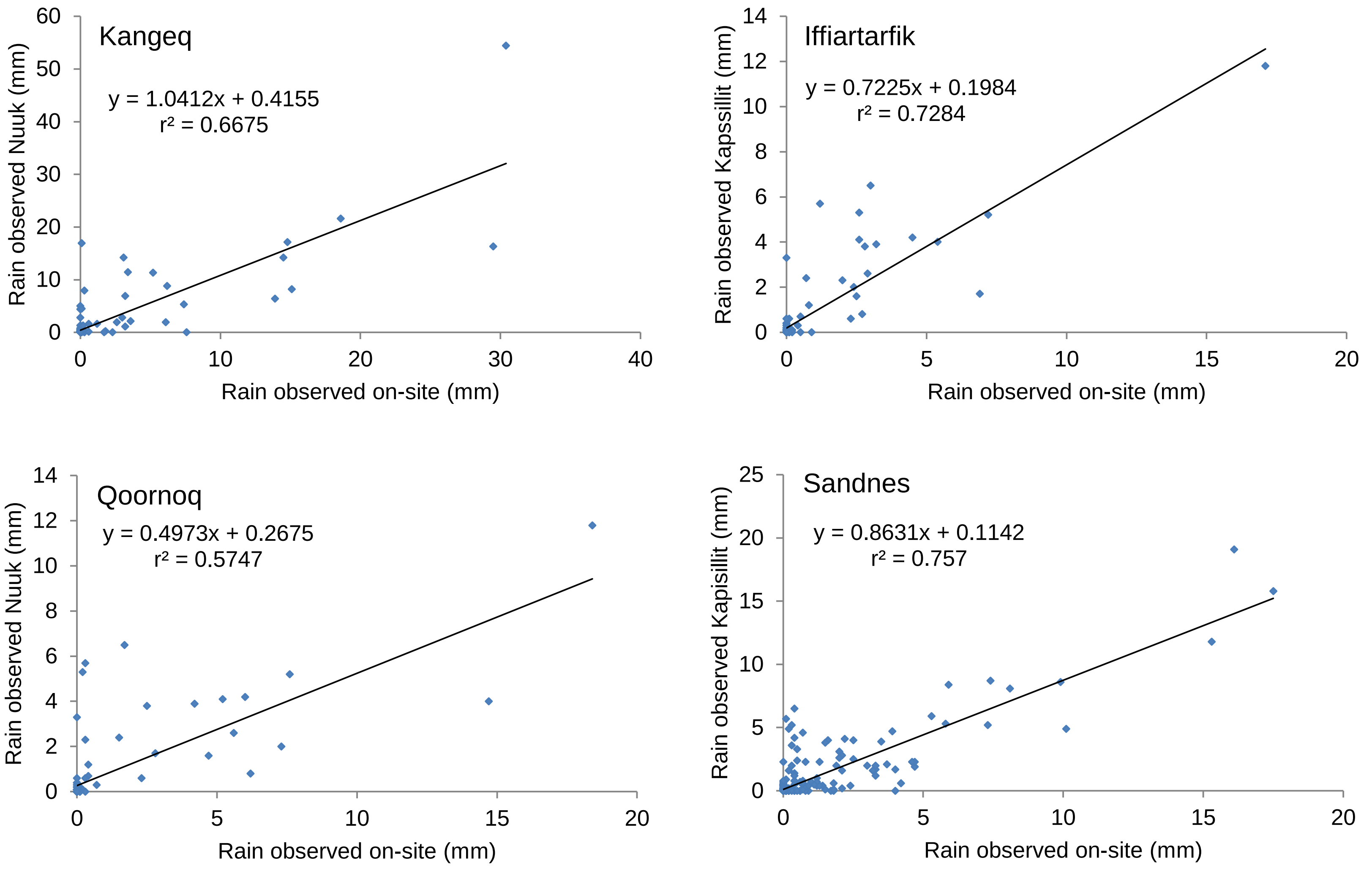

Supplementary Fig. S12: Linear regression between precipitation rates observed at the study sites in the frost-free period from 1st September 2016–31st July 2017 and daily precipitation sums from official meteorological stations in Nuuk and Kapisillit. Kangeq and Qoornoq correlated best with data from Nuuk and Iffiartarfik and Sandnes with data from Kapisillit.

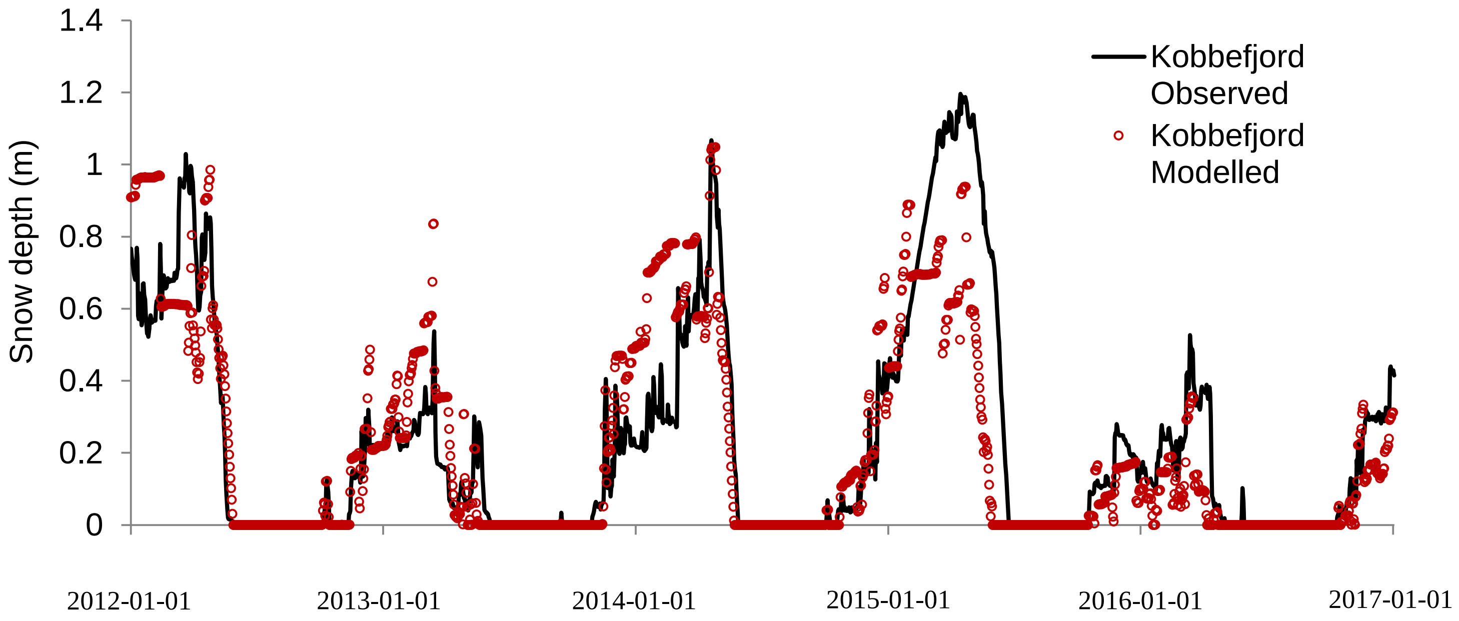

Supplementary Fig. S13: Simulated vs observed snow depths for the Nuuk Basic research station located in Kobbefjorden.

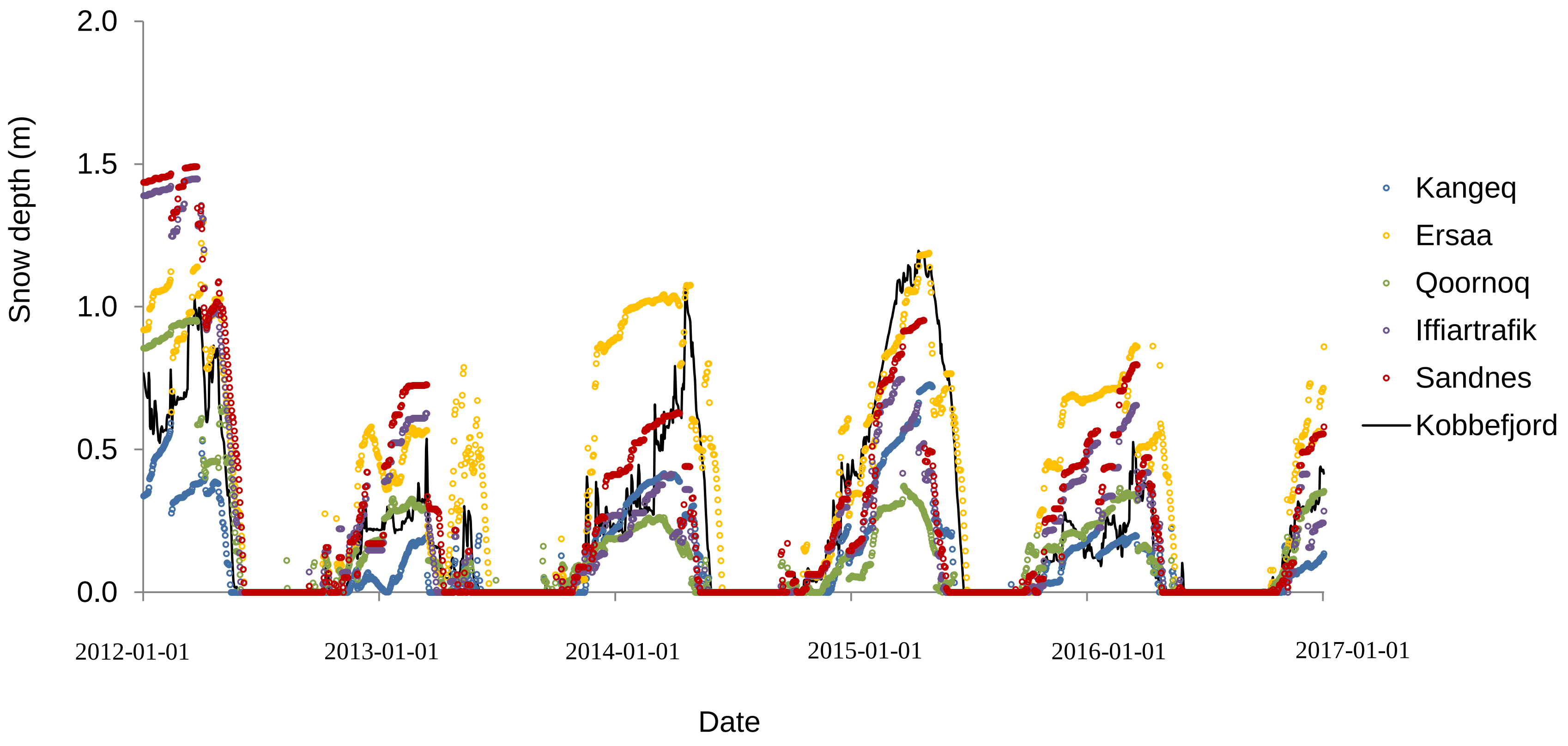

Supplementary Fig. S14: Modelled snow cover for the five study sites and the Nuuk Basic research station i Kobbefjord.

Supplementary Table S1: Study site informations. Samples were taken from the cultural phases highlighted in bold.

Site	Latitude	Longitude	Cultural phases
Kangeq	64°06.433' N	52° 03.104' W	Saqqaq, **Thule**
Ersaa	64°14.796' N	51° 36.449' W	**Thule**
Qoornoq	64°32.027' N	51°05.144' W	**Thule**
Iffiartarfik	64°27.567' N	50°38.738' W	**Norse**, Thule
Kilaarsarfik (Sandnes)	64°14.604' N	50° 10.510' W	Saqqaq, Dorset, **Norse**
V35 (Tummeralik)	64°17.196' N	50° 09.136' W	**Norse**
V53D	64°13.603' N	49° 49.157' W	**Norse**

Supplementary Table S2: The Q10 values obtained from O_2 consumption measurements at 1, 5, 10 and 15 ^oC.

Site	Depth	Q10 (1-15 ^oC)	r^2 (n=4)
Kangeq	20	2.6	0.99
Kangeq	30	2.3	0.97
Kangeq	40	2.2	0.92
Kangeq	50	2.1	0.96
Erssa	10	2.7	0.98
Erssa	20	2.3	0.95
Qoornoq	10	2.5	0.96
Qoornoq	20	2.3	0.93
Qoornoq	30	2.5	0.95
Qoornoq	40	2.1	0.93
Qoornoq	50	2.6	0.89
Iffiartarfik	10	2.0	0.99
Iffiartarfik	20	1.5	0.79
Iffiartarfik	30	1.7	0.99
Sandnes	20	1.7	0.69
Sandnes	40	2.4	0.95
Sandnes	60	2.5	0.97

Supplementary Table S3: Statistics on the agreement between observed (x) and simulated soil temperatures (y) for the calibration and test simulations.

	Calibration (Kangeq) 1 January 2015–31 August 2017				Calibration (Kangeq) 1 June–31 August 2015,2016 and 2017			
Depth (m)	N	r^2	Regression lines	Average (y-x)	N	r^2	Regression lines	Average (y-x)
0.1	974	0.86	y=0.99x-0.29	-0.29	276	0.74	y=0.95x+0.52	0.35
0.2	974	0.85	y=0.87x-0.15	-0.11	276	0.85	y=0.97x+0.01	0.01
0.4	974	0.78	y=0.99x-0.14	-0.13	276	0.90	y=1.26x-0.01	0.17
0.6	974	0.55	y=0.96x-0.13	-0.12	276	0.82	y=1.57x-0.03	-0.03

	Test 1 (Ersaa) 1 June–31 July 2017				Test 2 (Qoornoq) 1 June–31 August 2017			
Depth (m)	N	r^2	Regression lines	Average (y-x)	N	r^2	Regression lines	Average (y-x)
0.1	61	0.68	y=0.67x + 1.18	-0.08	92	0.60	y=1.20x - 1.24	-0.23
0.2	61	0.80	y=0.93x - 0.09	-0.28	92	0.79	y=0.95x - 0.35	-0.50
0.4	61	0.79	y=1.26x - 0.78	-0.25	92	0.82	y=0.99x - 0.05	0.03
	**Test 3 (Iffiartafik) 1 June–31 July 2017**				**Test 4 (Sandnes) 1 June–31 August 2017**			
Depth (m)	N	r^2	Regression lines	Average (y-x)	N	r^2	Regression lines	Average (y-x)
0.1	61	0.85	y=1.08x-0.41	0.07	92	0.39	y=0.73x+2.2	0.27
0.2	No data	No data	No data	No data	92	0.88	y=0.98x-0.03	-0.12
0.4	61	0.97	y=1.36x - 0.56	0.02	92	0.92	y=1.14x - 0.04	-0.13

Supplementary Table S4: Overview of the environmental parameters monitored at the five study sites and the equipment used.

Data type	Kangeq	Ersaa	Qoornoq	Iffiartarfik	Sandnes
**Air temperature**	CS215 Temperature & RH probe (Campbell Scientific)	*Data from an official meteorological station located 5 km to the South was used.	CS215 Temperature & RH probe (Campbell Scientific)	Tinytag (Gemini Data Loggers)	MP100A Temperature & RH probe (Campbell Scientific)
**Rain**	52202 Tipping Bucket Raingauge (Campbell Scientific)	*Data from an official meteorological station located 5 km to the South was used.	52202 Tipping Bucket Raingauge (Campbell Scientific)	52202 Tipping Bucket Raingauge (Campbell Scientific)	52202 Tipping Bucket Raingauge (Campbell Scientific)
**Snow cover**	Automatic camera		Automatic camera		Automatic camera
**Soil temperature**	107 temperature probes (Campbell Scientific)	Tinytag (Gemini Data Loggers)	107 temperature probes (Campbell Scientific)	Tinytag (Gemini Data Loggers)	107 temperature probes (Campbell Scientific)
**Soil moisture**	Theta Probe (Delta-T Devices Ltd, Cambridge, UK)	SM300 probe (Delta-T Devices Ltd, Cambridge, UK)	Theta Probe (Delta-T Devices Ltd, Cambridge, UK)	SM300 probe (Delta-T Devices Ltd, Cambridge, UK)	SM300 probe (Delta-T Devices Ltd, Cambridge, UK)
